# The second-to-fourth digit ratio correlates with aggressive behavior in professional soccer players

**DOI:** 10.3892/mmr.2013.1426

**Published:** 2013-04-10

**Authors:** VALENTINA PERCIAVALLE, DONATELLA DI CORRADO, MARIA CRISTINA PETRALIA, LINO GURRISI, SIMONA MASSIMINO, MARINELLA COCO

**Affiliations:** 1 Department of Sciences of Formation, Section of Physiology, University of Catania, Catania, Italy; 2 Department of Bio-Medical Sciences, Section of Physiology, University of Catania, Catania, Italy; 3 Department of Sport and Motor Science, University ‘Kore’ of Enna, Enna, Italy

**Keywords:** 2D:4D ratio, aggression, testosterone, soccer, playing positions

## Abstract

The aim of this study was to test the hypothesis that high levels of testosterone during prenatal life, testified by a low second-to-fourth digit ratio (2D:4D), as well as in adulthood affect the aggressive behavior of professional soccer players. Using 18 male professional players from a first level Italian Soccer Team we calculated: i) the 2D:4D ratio of the right hand, ii) the number of yellow and red cards per game, iii) the mean salivary testosterone concentration (Sal/T) and iv) the handling of aggressive impulses as assessed by the Picture Frustration test (PFT). Soccer players with a lower 2D:4D ratio had a higher number of fouls per game. A significant negative correlation was observed between Sal/T and 2D:4D ratio, as well as between 2D:4D ratio and the aggressiveness of players. By contrast, a significant positive correlation of Sal/T and fouls/game score and PFT was detected. No significant correlation was detected between 2D:4D or Sal/T and the playing position of players. Results of this study revealed that in professional soccer players, aggressive behavior, with the consequent increased risk of fouls during the game, is more likely to occur in individuals with high testosterone levels, not only in adulthood, but also during their intrauterine life.

## Introduction

At the 2006 FIFA World Cup in Germany, millions of people followed live the head-butt given by the French superstar Zinédine Zidane to the Italian defender Marco Materazzi.

Whether this aggressive behavior is the result of innate factors or acquired elements is a matter for debate. Animal studies have indicated a link between incidents of aggression and the individual level of circulating testosterone. However, results in relation to primates, particularly humans, are less clear cut and are at best only suggestive of a positive association in certain contexts (1).

It has been observed in male athletes that testosterone levels before a competition exhibit an increase that precedes the start of the competition and reflects the quality of the performance. In fact, testosterone levels measured in athletes with high-level performances are increased compared with those detected in low-level athletes (2).

It must be emphasized, nevertheless, that several studies in humans did not observe any correlation between testosterone and aggression (3–5). The existence of such a correlation could explain the so-called ‘roid rage’, which is the extremely aggressive behavior associated with the intake of large amounts of anabolic steroids (6,7). However, even if there is an effect of testosterone on aggression at extremely high doses, this does not necessarily suggest that testosterone is effective at physiological concentrations.

It has been suggested that prenatal androgens affect the developing brain by increasing its sensitivity to circulating testosterone later in life (8,9). These effects may include increased self-confidence (10), search persistence (11) and risk preferences (12–14), as well as intensified vigilance and quickened reaction times (15). A number of markers have been proposed for evaluating the effects of prenatal androgens (16), but the most suitable is likely to be the second-to-fourth digit length ratio (2D:4D), with a relatively longer fourth finger (i.e., lower 2D:4D ratio) indicating higher fetal androgen levels (17). However, a previous study (18) concluded that, due to the considerable within-group variability and between-group overlap, digit ratio is not a good marker of individual differences in prenatal androgen exposure. Recently, Manning (19) suggested that 2D:4D is determined not by prenatal androgens alone but by the balance of prenatal androgen to prenatal estrogen signaling in a narrow time window of fetal digit development. For ease of measurement and reproducibility, 2D:4D is used as a substitute measure for prenatal androgen exposure. Support for this use originates from the fact that digit growth and gonadal development are linked by the common influence of Hox genes (17,20). These findings suggest that sex steroids produced by the developing gonads exert significant modulatory effects on digit growth (21). Lower digit ratios have also been shown to correlate with increased sensitivity of androgen receptors (22) and human male reproductive function is negatively correlated with 2D:4D ratio (23). Moreover, 2D:4D ratios appear to be predictive of success among high-frequency financial traders (24), predicting entrance to and success in Medical Schools of state-run Italian Universities (25) and performance of competitive sports, such as basketball (26), skiing (27) and soccer (28).

Professional soccer players have lower 2D:4D ratios than controls. Soccer players in the 1st team squads have lower 2D:4D ratios than reserves or youth team players. Men who had represented their country had lower ratios than those who had not, and there was a significant (one-tailed) negative association between 2D:4D ratios and the number of international appearances after the effect of country was removed. These data suggest that prenatal and adult testosterone promotes the development and maintenance of traits that are useful in sports and athletics disciplines and in male:male fighting (28).

The present study was performed to test the hypothesis that not only high levels of testosterone in adulthood but also higher prenatal testosterone exposure may affect the aggressive behavior of professional soccer players. Specifically, we predicted that players with a lower 2D:4D ratio, due to their aggressive behavior, commit a high number of fouls during the match, punished by the referee by a caution (yellow card) or sending-off (red card).

To test our predictions, we recruited 18 male professional players from a professional soccer team of the ‘Series A’ Italian Football League. We used the numbers of yellow as well as red cards obtained by players as the primary measure of their aggressive behavior. We also measured the levels of salivary testosterone (Sal/T) in players and their aggression using the Picture Frustration test (PFT) by Rosenzweig (29).

## Materials and methods

### Participants

We recruited 18 male professional players from a first level Italian Soccer Team (Calcio Catania S.p.A.) which participated in the ‘Serie A’ championship 2010/2011 of the Italian Football League. The players received an introductory note that explained briefly that we were looking at the effects of prenatal testosterone on the shape of their right hand, but no information was provided about our hypothesis. Prior to providing a handprint, all the subjects completed a short questionnaire pertaining to their age, medical history and, in particular, whether they had broken the index or ring finger on their right hand. The participants also signed an informed consent form. Participants had a mean height of 1.78 m (±0.06), a mean body mass of 76.9 kg (±5.56) and a mean body mass index (BMI) of 24.2 (±0.53).

The number of played games, the number of min played in each game, and the number of yellow and red cards (fouls) were obtained for each player. In this way, the number of fouls/game (1 game = 90 min) was calculated.

The study was approved by the Ethics Committee of the Medical School at the University of Catania.

### Digit ratio measurement

The method used for measuring digit ratio was described by our group in a previous study (25). Briefly, to determine the 2D:4D ratio, we photocopied the players’ right hands and measured the digit length from the metacarpo-phalangeal crease to the finger tip. It has been observed that this crease appears around the 9th week of gestation and is one of the primary creases of the hand (30).

The 2D:4D ratio was determined from only the right hand, as the right-hand digit ratios exhibit more robust gender differences and appear to be more sensitive to prenatal androgens (31,32).

To measure the 2D:4D ratio we used practical recommendations suggested by Voracek *et al*(33) and those recently described by Coates and Hebert (34). In soft tissue, care must be taken to distinguish regular from irregular or secondary creases. Irregular creases form later than regular creases, after the 11th week of gestation when the fingers start to bend, disrupting the dermal surface (30,35). The handprints of the players were measured to determine 2D:4D ratio by one of the authors (M.C.) using calipers accurate to 0.2 mm.

### Hormone assessment

The method used for testosterone assessment was described by our group in a previous study (25). Briefly, saliva samples (1 ml) were collected at rest in sterile containers and stored at −80°C. Sugar-free gum (Vivident Xylit) was used to increase saliva flow in the participants (36). Since in adult males, the excretion of testosterone in saliva appeared to follow a circadian rhythm (37) and to be a pulsatile secretion (38), four samples were collected, at intervals of 30 min, between 9:00 and 1:00 a.m. Saliva was assayed using diagnostic kits (Diagnostic Systems Laboratories Inc., Webster, TX, USA) and modified radioimmunoassay methods (39). The testosterone assay sensitivity was 0.3 pg/ml, with intra-and inter-assay CVs of <9.2 and <8.3%, respectively. Only the highest value of the four measures obtained from each subject was used for the experiments.

### Picture frustration test

The method used for PFT assessment was described by our group in a previous study (25). Briefly, the Italian version of the PFT (29,40) permits an evaluation of the subject’s preferred way of handling aggressive impulses. The subject is asked to react verbally to 24 drawings showing common frustration situations by filling out an empty speech bubble for a character experiencing the frustration. The answers are assigned to 3 directions of aggression: ‘extraggression’, ‘intraggression’ and ‘imaggression’. The types of aggression include attending to the frustrating barrier (obstacle dominance), defending the organization of personality (ego-defense) or finding solutions (need-persistence), and some special indices. Descriptions of each factor are shown in Table I [based on (41)].

### Statistical analysis

Data were reported as the means ± SD. Data were collected and averaged, and then compared using unpaired Student’s t-test or one-way repeated measures ANOVA (Friedman test) followed by Dunn’s multiple comparison test. Correlation analysis was carried out by using one-tailed Pearson’s correlation analysis; significance was set at P<0.05. All analyses were performed using Systat Software Package version 11 (Systat Inc., Evanston, IL, USA). Statistical analysis was carried out according to guidelines for reporting statistics in journals published by the American Physiological Society (42).

## Results

In the present study, we first correlated the different aspects of aggression assessed by PFT with digit ratio. As shown in Table II, the only significant detected correlation was with extraggression-extrapersistive (P<0.05). The other extraggression scores, extrapeditive and extrapunitive, on the PFT test had no significant correlation with the 2D:4D ratio. Additionally, the intraggression and imaggression scores on the PFT test showed no significant correlation.

Table III shows data obtained for each soccer player, i.e., their playing position (goalkeeper, defender, midfielder or attacker), their 2D:4D ratio and Sal/T (pg/ml), their score in PFT test (extraggression-extrapersistive) and the number of fouls obtained per game.

**Figure 1:**
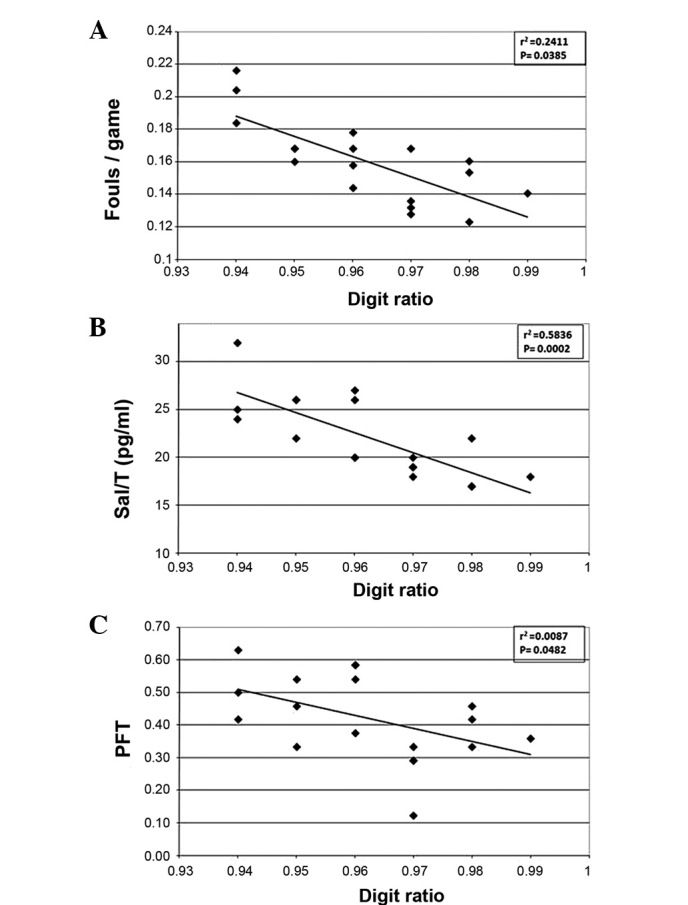
(A) Correlation between 2D:4D ratio and the number of fouls obtained by the player/game. (B) Correlation between 2D:4D ratio and mean salivary testosterone concentration (Sal/T) of each player. (C) Correlation between 2D:4D ratio and extrapersistive aggressiveness of each player.

Firstly, we analyzed the correlation between 2D:4D ratio and the number of fouls obtained by the player per game (Fig. 1A). As expected, players with a lower 2D:4D ratio had a higher number of fouls per game. In fact, the results showed that the lower a player’s 2D:4D ratio, the higher his fouls/game ratio (P=0.0385). Moreover, we studied possible correlations between the 2D:4D ratio of the players and their salivary testosterone concentration (Sal/T). Mean Sal/T of our players was 22.11 pg/ml (±4.19), indicating a significant negative correlation between the two variables (P=0.0002) (Fig. 1B). When the 2D:4D ratio of our players was plotted against their extrapersistive aggressiveness, one of the extraggression characteristics from the PFT (Fig. 1C), we observed a significant negative correlation between the two variables (P=0.0482). No correlation was observed between 2D:4D ratio and the role of the player in the team.

**Figure 2:**
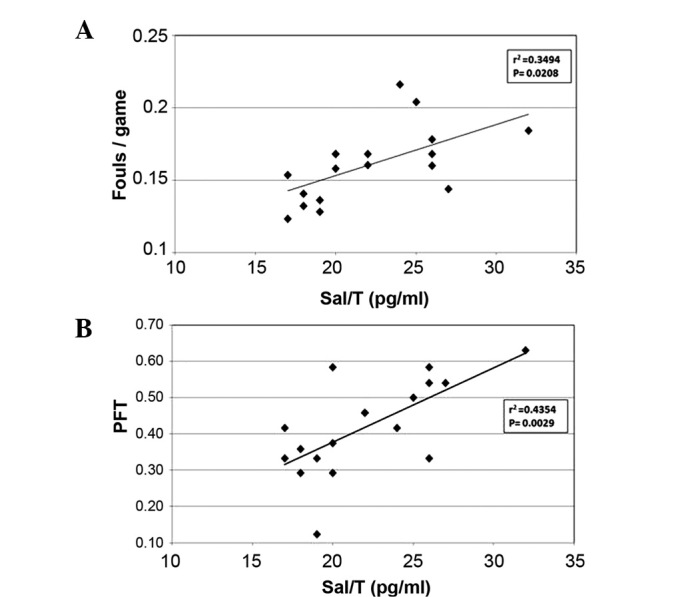
(A) Correlation between mean salivary testosterone concentration (Sal/T) values with fouls/game score of each player. (B) Correlation between Sal/T values with extrapersistive aggressiveness scores on the Picture Frustration test (PFT) of each player.

We also attempted to correlate the Sal/T values with fouls/game score and the extrapersistive aggressiveness scores on the PFT test. For the professional players included in this study, a significant positive correlation was observed between Sal/T and fouls/game score (P<0.01) and PFT (P<0.05) (Fig. 2). No significant correlation was detected between Sal/T and the playing position in our sample of soccer players.

## Discussion

By summarizing our results, we found a significant negative correlation between 2D:4D ratios in our sample of professional soccer players and their salivary testosterone levels, their extrapersistive aggressiveness (i.e., a mindset for which the solution to the frustrating situation is expected of someone else) and their number of fouls per game. No correlation was observed between 2D:4D ratio and the playing positions. Moreover, in the same players we detected a significant correlation between Sal/T and both the number of fouls per game and aggressiveness, but not with playing position.

Our results, beyond confirming the correlation between testosterone levels and 2D:4D ratios, provide support for the hypothesis that 2D:4D ratios predict a higher aggressive behavior and, therefore, a higher risk of fouls during the game.

Many investigators focus on the brain to explain aggression. Electrical stimulation of the hypothalamus causes aggressive behavior (43) and receptors that modulate aggression levels have been identified in the hypothalamus (44). These brain areas have direct connections with both the brainstem nuclei controlling vegetative functions, and with structures such as the amygdala and prefrontal cortex.

Stimulation of the amygdala results in augmented aggressive behavior in hamsters (45,46), while in rhesus monkeys, neonatal lesions in the amygdala or hippocampus result in reduced expression of social dominance, related to the regulation of aggression and fear (47). In many mammals, the circuitry within the amygdala appears to be involved in the control of aggression. However, the role of the amygdala is less clear in primates and appears to depend more on situational context, with lesions leading to increases in either social affiliatory or aggressive responses.

The prefrontal area of the cerebral cortex is involved in aggression, along with many other functions, including inhibition of emotions. Reduced activity of the prefrontal cortex, in particular its medial and orbitofrontal portions, has been associated with violent/antisocial aggression (48).

In a previous study, a significant correlation between 2D:4D ratios and salivary testosterone in adult men was noted (49). However, in the same study, no significant correlation was observed between digit ratio and reactive aggression. The discrepancy may be due to the different methods used for measuring aggression: in the present study we used a validated and widely used test (PFT), whereas Benderlioglu and Nelson (49) utilized an adaptation of the Kulik and Brown (50) method, which is less widespread and not yet validated.

The correlation between baseline testosterone concentrations and aggressiveness, detected in the present study, has been previously observed (25,51–53), although other investigators have failed to replicate this finding (54,55). These contradictory data for aggression may be due, in part, to the use of self-report measures as opposed to the direct measurement of aggressive behavior (56). Additionally, circadian rhythm (37), pulsatile secretion (38) or dynamic fluctuations (57) in testosterone concentrations may be more related to aggressive behavior than mean daily testosterone concentrations. To minimize these possible errors, for the evaluation of aggression, we directly measured aggression of our players by using one of the most commonly used tests (PFT) for this purpose, while testosterone collection was carried out between 9:00 and 12:00 a.m., and 4 samples were collected, at intervals of 30 min. Only the highest value of the four measures obtained from each subject was used for the present experiments.

In conclusion, this study appears to show that, in professional soccer players, aggressive behavior with the consequent increased risk of fouls during the game, is more likely in individuals with high testosterone levels, not only in adulthood, but also during their intrauterine life.

## Figures and Tables

**Table I:** Constructs of reaction to frustration.^a^

	Type of aggression		
	
Direction of aggression	Obstacle-dominance (O-D)	Ego-defence (etho-defense) (E-D)	Need-persistence (N-P)
Extragression (E-A)	E^1^ (Extrapeditive): The presence of the frustrating obstacle is (insistently) pointed out.	E (Extrapunitive): Blame, hostility are turned against some person or thing in the environment. E (a variant of E): subject denies that he is responsible for some offense with which he is charged.	e (Extrapersistive): A solution to the frustrating situation is expected of someone else.
Intraggression (I-A)	I^1^ (Intropeditive): Obstacle is construed as non-frustrating even beneficial; subject can also emphasize his embarrassment for causing another person’s frustration	I (Intropunitive): Blame, censure are directed by the subject - upon himself. I (a variant of I): subject admits his guilt but denies any essential fault by referring to unavoidable circumstance.	i (Intropersistive): Amends are offered by the subject, usually from a sense of guilt, to solve the problem.
Imaggression (M-A)	M^1^ (Impeditive): Obstacle of frustration is minimized almost to the point of denying its existence.	M (Impunitive): Blame for the frustration is evaded altogether, the situation is regarded as unavoidable; the frustrating individual is absolved.	m (Impersistive): Hope is expressed that time or normally expected circumstances will bring about a solution of the problem; patience and conformity are characteristic.

a Previously shown in ref (25).

**Table II:** Correlation between RPF scores and 2D:4D ratio.

	RPF test	P-value
Intraggression	Intropeditive	NS
	Intropunitive	NS
	Intropersistive	NS
Imaggression	Impeditive	NS
	Impunitive	NS
	Impersistive	NS
Extraggression	Extrapeditive	NS
	Extrapunitive	NS
	Extrapersistive	**<0.05**

RPF, Rosenzweig picture frustration test; NS, non-significant. Bold, statistically significant.

**Table III:** Data obtained for the 18 professional soccer players.

Players	Role	2D:4D	Sal/T (pg/ml)	PFT (E-A) e	Fouls/game
1	G	0.99	18	0.36	0.14
2	D	0.94	24	0.42	0.22
3	D	0.98	17	0.42	0.15
4	M	0.98	22	0.46	0.16
5	M	0.96	26	0.58	0.18
6	D	0.94	32	0.63	0.18
7	D	0.96	20	0.38	0.17
8	D	0.98	17	0.33	0.12
9	M	0.97	19	0.33	0.13
10	G	0.96	27	0.54	0.14
11	A	0.95	26	0.54	0.17
12	G	0.95	26	0.33	0.16
13	A	0.97	18	0.29	0.13
14	D	0.97	20	0.29	0.17
15	D	0.97	19	0.12	0.14
16	D	0.95	22	0.46	0.17
17	M	0.94	25	0.50	0.20
18	A	0.96	20	0.58	0.16
**Mean**		**0.96**	**22.11**	**0.42**	**0.16**
**SD**		**0.02**	**4.19**	**0.13**	**0.03**

2D:4D, second-to-fourth digit ratio of the right hand; Sal/T, mean salivary testosterone concentration; PFT (E-A) e, score in PFT test (extraggression - extrapersistive); fouls/game, number of fouls per game (1 game = 90 min); G, goalkeeper; D, defender; M, midfielder; A, attacker. Bold, ?
